# Physicians’ clinical prediction of survival in head and neck cancer patients in the palliative phase

**DOI:** 10.1186/s12904-020-00682-2

**Published:** 2020-11-24

**Authors:** Arta Hoesseini, Marinella P. J. Offerman, Bojou J. van de Wall-Neecke, Aniel Sewnaik, Marjan H. Wieringa, Robert J. Baatenburg de Jong

**Affiliations:** 1 Department of Otorhinolaryngology and Head and Neck Surgery, Erasmus MC Cancer Institute, Erasmus University Medical Center, Dr. Molewaterplein 40, 3015 GD Rotterdam, The Netherlands; 2 Department of Education and Research, Elisabeth TweeSteden Hospital, Tilburg, The Netherlands

**Keywords:** Prognosis, Palliative care, Head and neck cancer, Prediction, Survival, Counseling

## Abstract

**Background:**

The prognosis of patients with incurable head and neck cancer (HNC) is a relevant topic. The mean survival of these patients is 5 months but may vary from weeks to more than 3 years. Discussing the prognosis early in the disease trajectory enables patients to make well-considered end-of-life choices, and contributes to a better quality of life and death. However, physicians often are reluctant to discuss prognosis, partly because of the concern to be inaccurate. This study investigated the accuracy of physicians’ clinical prediction of survival of palliative HNC patients.

**Methods:**

This study was part of a prospective cohort study in a tertiary cancer center. Patients with incurable HNC diagnosed between 2008 and 2011 (*n* = 191), and their treating physician were included. Analyses were conducted between July 2018 and February 2019. Patients’ survival was clinically predicted by their physician ≤3 weeks after disclosure of the palliative diagnosis. The clinical prediction of survival in weeks (CPS) was based on physicians’ clinical assessment of the patient during the outpatient visits. More than 25% difference between the actual survival (AS) and the CPS was regarded as a prediction error. In addition, when the difference between the AS and CPS was 2 weeks or less, this was always considered as correct.

**Results:**

In 59% (*n* = 112) of cases survival was overestimated. These patients lived shorter than predicted by their physician (median AS 6 weeks, median CPS 20 weeks). In 18% (*n* = 35) of the cases survival was correctly predicted. The remaining 23% was underestimated (median AS 35 weeks, median CPS 20 weeks). Besides the differences in AS and CPS, no other significant differences were found between the three groups. There was worse accuracy when predicting survival closer to death: out of the 66 patients who survived 6 weeks or shorter, survival was correctly predicted in only eight (12%).

**Conclusion:**

Physicians tend to overestimate the survival of palliative HNC patients. This optimism can result in suboptimal use of palliative and end-of-life care. The future development of a prognostic model that provides more accurate estimates, could help physicians with personalized prognostic counseling.

## Background

HNC patients in general have a poor prognosis. The five-year survival rate varies between 30 and 70%, depending on the stage and location of the tumor [1]. Consequently, HNC treating physicians are regularly confronted with patients entering the palliative phase. The survival of patients with incurable HNC is short, with a mean of 5 months which can range from days to more than 3 years [2]. In our institute, we define the palliative phase as beginning at the moment of diagnosis of an incurable head and neck tumor or when the patient declines curative treatment [2, 3]. A head and neck tumor can be incurable for several reasons: inoperability plus no other curative treatment options, distant metastasis, the presence of severe comorbidity, and/or poor performance status of the patient.

Adequate counseling in the palliative phase requires an insight of what and when can be expected during the course of disease. HNC in this phase can cause specific end-of-life issues because of its local anatomy and the consequences of treatment. Examples are airway complications, communication difficulties, dysphagia, facial disfigurement, neuropathic pain, and psychosocial complaints [4, 5]. Given the short length of the palliative phase, discussing prognosis early in the disease trajectory enables patients to make well-considered end-of-life choices which could contribute to a better quality of life (QoL), and quality of death (QoD) [6]. Previous research has shown that patients, caregivers, and physicians have different views on a “good death” [7, 8]. Although there is no clear definition of the concept, there are some recurrent themes derived from qualitative research among terminal patients with and without cancer. A recently published systematic review that focused on patients’ perspectives on a “good death” identified the following core elements: control of pain and symptoms, clear decision making, feeling of closure, being seen and perceived as a person, preparation for death, and being still able to give something to others [8]. A “good death” is based on individual preferences and shaped by culture, religion, age, disease, financial status and life circumstances [8].

Talking about death and asking patients in the palliative phase what they consider to be a “good death” could also help to identify goals for end-of-life care [9]. Earlier studies showed that patients who have discussed end-of-life care with their physician, are less likely to receive burdensome care, like chemotherapy, and more likely to receive hospice care [10, 11]. Adequate timing of counseling is crucial and therefore a reliable prediction of the remaining life-span can be valuable information for patients. Furthermore, various studies have shown incurable patients’ desire for detailed prognostic information [12–14]. This allows them to prepare themselves and their families for what’s coming, and assist in their end-of-life decisions [13]. Consequently, end-of-life discussion are an important part of oncologists’ work. However, physicians often feel uncomfortable and reluctant to discuss prognosis in the palliative phase, partly because of the concern to potentially being proved inaccurate [15]. In addition, doctors’ natural impulse is to treat, while a palliative patients desire may be different after realistic prognostic disclosure.

Are doctors’ worries about inaccurate estimations of survival legitimate? Previous studies on prognostic accuracy in palliative care are heterogeneous and none of these studies focused on HNC specifically [16, 17]. This study set out to examine the accuracy of physicians estimations of survival of palliative HNC patients.

## Methods

### Study design

Data were collected during a prospective cohort study, approved by our medical ethical committee [MEC 2008–133]. During this study palliative patients with histologically proven squamous cell carcinoma of the head and neck were eligible for inclusion. Patients’ palliative diagnosis was discussed in our tumor board. Subsequently, the patients were informed the next week. Hereafter, patients’ survival was predicted by their treating physician by reporting how long they thought the patient would live (open text field). This clinical prediction of survival (CPS) was based on physicians’ clinical assessment of the patient during the outpatient visits. These estimations were physicians’ best guesses about the remaining life-span of these patients (physician-recalled). Information about survival was only communicated to patients on their request. Analyses were conducted between July 2018 and February 2019.

### Eligibility criteria

**Fig. 1:**
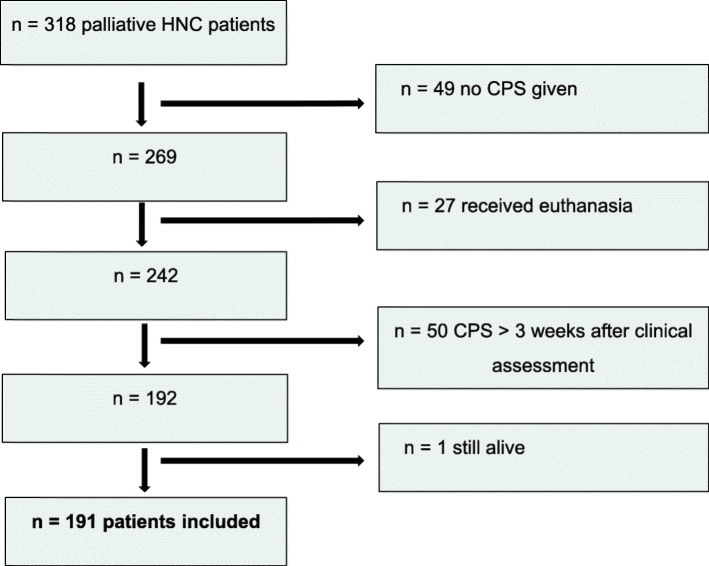
Flowchart summarizing inclusion and exclusion criteria. CPS = Clinical prediction of survival

Figure 1 shows a flowchart of patient inclusion. Palliative patients with histologically proven squamous cell carcinoma of the head and neck who were seen in the Erasmus MC Rotterdam Cancer Institute from October 2008 until October 2011 were eligible for inclusion (*n* = 318). Subsequently, in 269 patients survival was predicted by their physician. Of the 269 patients, 27 received euthanasia and were excluded. Patients given palliative sedation (*n* = 88) were not excluded, as the goal of palliative sedation is to relieve suffering, and not to shorten patients life [18]. Survival was predicted by their physician shortly before or after the outpatient visit in which the palliative diagnosis was communicated. In 50 cases survival was predicted more than 3 weeks after this outpatient visit. To limit recall bias of the clinical assessment, these 50 patients were excluded. In the remaining group one patient was still alive at the end of follow-up and was also excluded. The clinical prediction of survival (CPS) in the remaining 191 patients was analyzed. Patient and tumor data were collected from the electronical patient file. The majority of predictions was made by eight head and neck surgeons (92.1%, *n* = 176), followed by two radiotherapists (7.9%, *n* = 15), with varying levels of practice experience.

### Definitions

- *Palliative phase* was defined as beginning at the moment of diagnosis of an incurable head and neck tumor or when the patient declines curative treatment (*palliative diagnosis*). A head and neck tumor can be incurable for several reasons: inoperability plus no other curative treatment options, distant metastasis, the presence of severe comorbidity, and/or poor performance status of the patient.
- *Actual survival* (AS) was defined as the period in weeks between the consultation in which the palliative diagnosis was communicated and the actual date of death.
- *Clinical prediction of survival* (CPS) was defined as the period in weeks between the consultation in which the palliative diagnosis was communicated and the predicted date of death.
- The *survival difference* (SD) was defined as the time in weeks between the AS and the CPS.
- The *prediction error* was defined as > 25% survival difference between the AS and the CPS.

### Institutional routine

In our institution, patients with head and neck cancer are evaluated by the head and neck surgeons and the radiotherapists. After diagnostics, patients are discussed in our multidisciplinary tumor board. In this weekly board meeting, medical oncologists, head and neck surgeons, radiotherapists, radiologists, geriatricians, and physician assistants are present to discuss all patients with a HNC diagnosis, both curative and palliative. After the board meeting, the responsible physician discusses the boards’ recommendations with the patient during a patient encounter as soon as possible. When either the board or the patient decide to pursue a palliative trajectory, patients are referred to our Expert Center for Palliative Care. In 2005 we have set up this Center for patients with HNC and their families or significant others, aiming to improve the quality of life in the palliative phase [19]. The Expert Center team consist of: a dedicated head and neck cancer surgeon acting as a clear contact person for patients, specialist nurses, psychologists, speech therapists, a pain team including anesthesiologists, a dietician, social workers, and representatives of the religious profession. The specialized nurses provide information and psychosocial support to patients and relatives, handle pain management, and screen psychosocial needs and other relevant data for effective allocation of specialized care [19]. They also contact general practitioners (GP), as GP’s rarely see HNC cases in their daily practices. These nurses play a crucial role by ensuring more efficient and effective communication between physician, patient, and other caregivers [19]. Since 2016, patients are also monitored using a validated questionnaire: EORTC QLQ-C15-PAL [20], which measures quality of life and functioning in the palliative phase. As patients may be too fragile to visit the hospital, this is often done by telephone.

### Clinical prediction of survival groups

We defined three prediction groups:

1. *Correct prediction*: ≤ 25% difference between the AS and the CPS. In addition, when the difference between the AS and the CPS was 2 weeks or less, this was always considered as correct.
2. *Underestimation*: the AS was > 25% longer than the CPS, i.e. patients lived longer than predicted.
3. *Overestimation*: the AS was > 25% shorter than the CPS, i.e. patients lived shorter than predicted.

### Statistical analysis

Statistical analysis was done using Statistical Package for the Social Sciences (SPSS) statistics version 25. All tests were 2 sided with *P* < 0.05 as the limit of statistical significance. Tests used for continuous variables were the Independent-Samples Mann-Whitney U test, and the Independent-Samples Kruskal-Wallis test. The Pearson χ2 test was used for categorical variables. Overall survival function was analyzed using the Kaplan-Meier method. Statistical significance was assessed using the log-rank test.

## Results

### Patient characteristics

Patient characteristics of both the included and excluded group are shown in Table 1. There were no missing data. No significant differences were found between the excluded and included group. In total 191 (60%) out of 318 patients were included. The patient who was still alive at follow-up had an inoperable T4N0M0 squamous cell carcinoma of the maxillary sinus and was treated with palliative radiotherapy consisting of 16 fractions of 3.13 Gy, with a total dose of 50.08 Gy.

**Table 1:** Patient characteristics

	Included	Excluded	*P* Value
No. of patients	191	127	–
Median age, years (Q1 – Q3)	64.0 (57.0–76.0)	64.0 (55.0–69.0)	0.142
Age range, years	23–100	42–91	–
Sex			
Men	138 (72.3%)	96 (75.6%)	0.508
Women	53 (27.7%)	31 (24.4%)	
Tumor localization			
Lip	0	1 (0.8%)	
Oral cavity	54 (28.3%)	35 (27.6%)	
Oropharynx	54 (28.3%)	40 (31.5%)	
Nasopharynx	4 (2.1%)	5 (3.9%)	–
Hypopharynx	26 (13.6%)	15 (11.8%)	
Larynx	33 (17.3%)	17 (13.4%)	
Nasal cavity	3 (1.6%)	5 (3.9%)	
Maxillary sinus	5 (2.6%)	2 (1.6%)	
Salivary gland	2 (1.0%)	0	
Unknown primary	10 (5.2%)	7 (5.5%)	
Tumor stage			
I - III	15 (7.9%)	16 (12.5%)	
IVa	55 (28.8%)	41 (32.3%)	–
IVb	31 (16.2%)	15 (11.8%)	
IVc	90 (47.1%)	55 (43.3%)	
ACE-27			
0	46 (24.1%)	31 (24.4%)	
1	61 (31.9%)	31 (24.4%)	0.401
2	53 (27.7%)	37 (29.1%)	
3	31 (16.2%)	28 (22.0%)	
Cause incurable disease			
No curative treatment possible	172 (90.1%)	105 (82.7%)	0.055
Patients’ choice	19 (9.9%)	22 (17.3%)	
Palliative sedation	68 (35.6%)	32 (25.2%)	0.050
Euthanasia	0	27 (21.3%)	–
Carotid blowout syndrome	9 (4.7%)	5 (3.9%)	–
Suicide	0	1 (0.8%)	–
Median survival, weeks (95% CI)	12.0 (9.3–14.7)	12.0 (7.7–16.3)	0.753

### Clinical prediction of survival

**Fig. 2:**
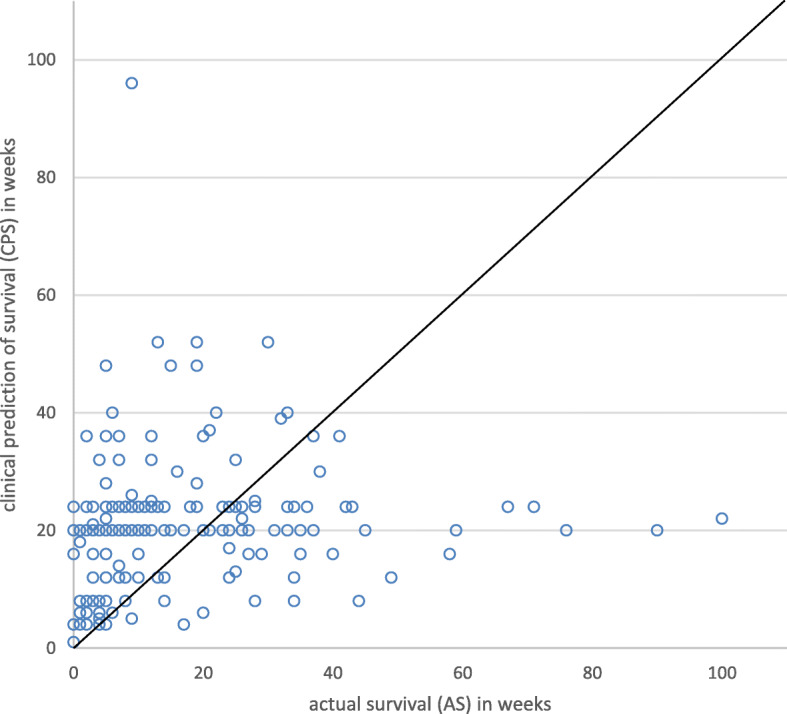
Clinical prediction of survival (CPS) versus actual survival (AS) for each individual. Each point represents a patient. Points around the 45 degree line represent patients who lived as long as predicted, points above the line represent patients who lived shorter than predicted, and points below the line represent patients who lived longer than predicted. Pearson correlation coefficient of 0.042 (*p* = 0.568). Four outliers are not shown (AS 220; 194; 192; 183 weeks versus a CPS of 20; 20; 12; 20 weeks)

**Fig. 3:**
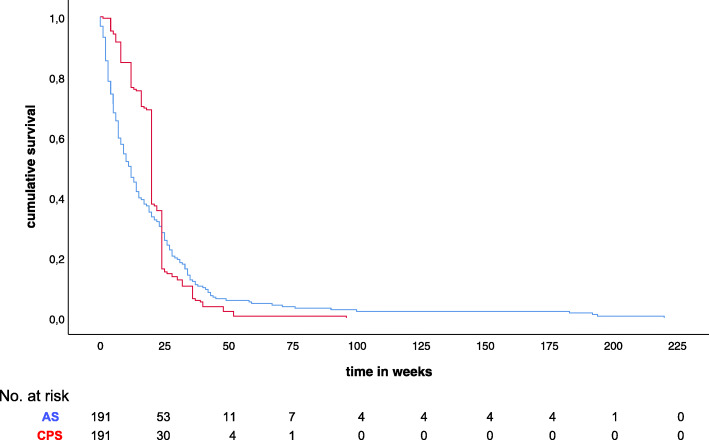
Overall survival function of the actual survival (AS, blue curve) versus the clinical prediction of survival (CPS, red curve) in all patients

Figure 2 shows a scatter plot of the CPS versus the AS. Each point represents a patient. Figure 3 shows the overall survival function of the AS and CPS of all patients (*p =* 0.124). Characteristics of the different survival prediction groups are shown in Table 2. In only 18% (*n* = 35) of cases survival was correctly predicted, while 59% (*n* = 112) of patients lived shorter than predicted (overestimation). The remaining 23% lived longer than predicted (underestimated). Variables with missing data were: *n* = 4 marital status (2.1%), n = 4 smoking (2.1%), *n* = 5 alcohol (2.6%), *n* = 26 no. alcohol units / day (13.6%), and *n* = 37 Body Mass Index (BMI) (19.4%). The AS and CPS differed significantly between groups. No other significant differences were found between groups. Out of the 66 patients who survived 6 weeks or shorter, survival was correctly predicted in only eight (12%) patients and overestimated in the remaining 58 (88%) patients.

**Table 2:** Characteristics of the prediction groups

	Overestimation^a^	Correct	Underestimation^a^	*P* Value
No. of patients	112 (58.6%)	35 (18.3%)	44 (23.0%)	
Median age, years (Q1– Q3)	65.0 (58.0–76.8)	61.0 (56.0–77.0)	64.0 (54.3–75.5)	0.627
Sex				
Men	80 (71.4%)	25 (71.4%)	33 (75.0%)	0.898
Women	32 (28.6%)	10 (28.6%)	11 (25.0%)	
Tumor localization				
Lip	0	0	0	
Oral cavity	34 (30.4%)	10 (28.6%)	10 (22.7%)	
Oropharynx	28 (25.0%)	9 (25.7%)	17 (38.6%)	
Nasopharynx	1 (0.9%)	2 (5.7%)	1 (2.3%)	
Hypopharynx	14 (12.5%)	5 (14.3%)	7 (15.9%)	–
Larynx	24 (21.5%)	4 (11.5%)	5 (11.3%)	
Nasal cavity	2 (1.8%)	1 (2.9%)	0	
Maxillary sinus	4 (3.6%)	1 (2.9%)	0	
Salivary gland	1 (0.9%)	1 (2.9%)	0	
Unknown primary	4 (3.6%)	2 (5.7%)	4 (9.1%)	
Tumor stage				
I-III	7 (6.3%)	2 (5.7%)	6 (13.6%)	
IVa	34 (30.4%)	6 (17.1%)	15 (34.1%)	–
IVb	18 (16.1%)	8 (22.9%)	5 (11.4%)	
IVc	53 (47.3%)	19 (54.3%)	18 (40.9%)	
ACE-27^b^				
0 (none)	25 (22.3%)	10 (28.6%)	11 (25.0%)	
1 (mild)	41 (36.6%)	6 (17.1%)	14 (31.8%)	0.409
2 (moderate)	31 (27.7%)	10 (28.6%)	12 (27.3%)	
3 (severe)	15 (13.4%)	9 (25.7%)	7 (15.9%)	
Marital status				
Alone	46 (41.8%)	14 (42.4%)	22 (50.0%)	0.642
Married / partner	64 (58.2%)	19 (57.6%)	22 (50.0%)	
Smoking				
Current / past	96 (87.3%)	29 (87.9%)	37 (84.1%)	0.848
No	14 (12.7%)	4 (12.1%)	7 (15.9%)	
Alcohol				
Current / past	88 (80.7%)	24 (72.7%)	39 (88.6%)	0.206
No	21 (19.3%)	9 (27.3%)	5 (11.4%)	
Median no. alcohol units / day (Q1 – Q3)	2.0 (0.3–6.0)	3.0 (0–5.0)	3.0 (1.0–4.0)	0.925
Median BMI^c^ (Q1-Q3)	20.9 (17.9–23.9)	20.4 (18.4–22.8)	21.2 (18.6–24.8)	0.716
Palliative sedation	36 (32.1%)	11 (31.4%)	21 (47.7%)	0.159
Carotid blowout syndrome	7 (6.3%)	1 (2.9%)	1 (2.3%)	–
Median survival, weeks (95% CI)				
Actual survival	6.0 (4.8–7.2)	23.0 (19.1–6.9)	35.0 (32.1–37.9)	0.000
Clinical Prediction of Survival	20 (19.5–20.5)	20 (12.8–27.2)	20 (18.9–21.1)	0.002

^a^Overestimation: patients lived shorter than expected, actual survival is > 25% shorter than the clinical prediction of survival. Underestimation: patients lived longer than expected, actual survival is > 25% longer than the clinical prediction of survival

^b^ Adult Comorbidity Evaluation 27

^c^ Body Mass Index

## Discussion

This study set out to examine the accuracy of physicians’ clinical prediction of survival in palliative HNC. Survival predictions were accurate in less than one out of five palliative HNC patients (18%), while 59% of the predictions were overoptimistic, meaning that patients lived shorter than predicted by their physician. This overestimated group had the worst actual survival with a median of 6 weeks. Furthermore, we found, in agreement with earlier research, worse accuracy when predicting survival closer to death [21]: out of the 66 patients who survived 6 weeks or shorter, survival was correctly predicted in only 12%.

Our results were comparable with previous research among other cancer types that also showed physicians’ tendency to overestimate [16, 17, 21–24]. Several factors may be associated with a lower prognostic accuracy. First, the level of physicians’ knowledge on mean survival rates in palliative HNC patients could have caused some optimism in their clinical prediction. This knowledge is partly based on a previously reported mean survival rate of 5 months [2], while the current study shows a median survival of only 3 months. This former reported more favorable survival could be due to the inclusion of patients with skin cancer (5.7%), histology other than squamous cell carcinoma (10.3%), and including less patients with severe comorbidity, defined by an Adult Comorbidity Evaluation 27 (ACE-27) score of 3 (9.5% versus 18.6% in the current study). Another factor that could be associated with lower prognostic accuracy is the duration of the doctor-patient relationship: the longer the doctor knows the patient, the lower the prognostic accuracy [22]. Doctors are trained to act, solve problems, and treat patients. Being optimistic and overestimating survival could therefore be a strategy to maintain hope [25]. Although this strategy seems plausible, evidence suggests that hope is maintained by the truth [26–28]. In an advanced cancer population, patients who were given a poor prognosis in terms of survival and QoL, low chance of response to treatment, and no chance of cure remained hopeful about their future [26–28]. Not only do physicians tend to overestimate survival, patients are also prone to do so [29, 30]. This could be due to misinterpretation or lack of information, as patients often do not receive prognostic information from their physicians [31–34]. Patients’ denial affects misunderstanding the prognosis and goals of treatment [35, 36]. Although patients often do not receive prognostic information, they generally have high levels of information need about life expectancy and they want at least some discussion of this topic at the time of diagnosis or shortly after [37]. While some patients seek qualitative information about the prognosis, for example: *is the illness curable?*, others prefer quantitative information like survival rates or other statistics [14, 36, 38]. Curatively treated HNC patients that participated in our focus group research had a stronger preference for quantitative prognostic information in the hypothetical case of cancer recurrence and a poor prognosis [36]. Besides meeting patients’ information needs, providing prognostic information in the palliative phase can affect patients’ end-of-life choices. An accurate estimation of survival makes it possible to communicate a more realistic indication of the end-of-life with the patient. Consequently, physicians and their palliative team can optimize palliative care planning. This may also have an impact on decisions about palliative treatment or involvement in clinical trials [10, 11, 29]. Multiple studies have shown that with palliative or hospice care survival is not reduced, but equal or even prolonged [26, 39, 40]. Temel et al. randomly assigned 151 patients with metastatic non–small-cell lung cancer to: 1) usual oncology care or 2) usual oncology care plus early palliative care. Results showed that the group that received early palliative care had significant better QoL, less depressive symptoms and lived longer while they received less aggressive treatment [39]. Another study among 4493 terminal patients found that hospice care significantly extended survival in patients with lung and pancreatic cancer [40].

### Strengths and limitations

A major strength of this study is the paucity of research on this topic among palliative HNC patients. Furthermore, we asked physicians to give a clinical prediction at the moment that this was highly relevant to the patient: shortly after disclosing the palliative diagnosis. Previous studies among other patient groups are often heterogeneous of design and include estimations that are given down the road instead of at the beginning of the palliative phase. However, we also excluded 50 cases because survival was predicted more than 3 weeks after the outpatient visit in which the palliative diagnosis was discussed. The reason for exclusion was to limit doctors’ recall bias. Although this 3 week cut-off point leads to data loss, no significant differences were found between the inclusion and exclusion group. Another limitation of this study is the number of available variables to compare the prediction groups.

### Clinical implications & future research

Our results show that HNC treating physicians tend to overestimate survival. This can result in suboptimal use of palliative and end-of-life care. Discussing prognosis as soon as possible in the disease trajectory, enables patients to make well-considered end-of-life choices and prepare for a good death. We hope that sharing these results will make HNC treating physicians become more aware of their tendency to overestimate survival. Future research could aid to fill this gap by developing a prognostic model that predicts survival more accurately. Such a model could help physicians’ to disclose more accurate prognostic information to guide their discussions with patients and caregivers. Although many prognostic models have been developed, few are actually used in clinical practice [41, 42]. One of the key factors for successful implementation is whether a model is supported by professionals in the field in question [41]. Previous research by Hallen et al. showed physicians’ willingness to use prognostic models in end-of-life care, aiming to improve prognostic confidence [15]. It also enabled them to take a more directive role in specific cases where the expected prognosis significantly differed from patients’ expectations [15]. Also, in case of conflicting opinions about prognosis, especially among physicians, it was thought to be helpful and reduce ambiguity [15]. We recently updated our prognostic model “OncologIQ” and added new prognostic factors [43, 44]. OncologIQ has been developed to support shared decision making (SDM) in patients with primary HNC that are eligible for curative treatment. The model calculates the 1- to 10-year overall survival based on several prognostic factors like age, sex, comorbidity, and socioeconomic status [45–47]. Currently, we are developing a similar prognostic model for palliative HNC patients. Hopefully, this model will take away some reluctance to discuss the prognosis in the palliative phase, and give rise to more realistic prognostic discussions. Due to the implementation of our Healthcare Monitor [48] we are routinely collecting data on the QoL and functioning in the palliative phase, using the EORTC QLQ-C15-PAL [20]. These data can be used in future prognostic research to model QoL. Indeed information on prognosis is not a stand-alone concept and future research should focus on how to share this information with palliative patients’ and their caregivers during patient-clinician discussions [49].

## Conclusions

This study addresses the difficulty of providing an accurate survival estimation of HNC patients in the palliative phase. It is an important topic to study as patients in this phase often desire accurate prognostic information. Discussing this as soon as possible in the disease trajectory, enables patients to make well-considered end-of-life choices and prepare for a good death. We hope that physicians treating HNC will become more aware of their tendency to overestimate survival, as this optimism can result in suboptimal use of palliative and end-of-life care. The future development of a prognostic model for incurable HNC patients, could help physicians with personalized prognostic counseling.

## Data Availability

The datasets generated and/or analyzed during the current study are not publicly available. The full dataset could contain information that might compromise research participants’ privacy and/or their conditions of consent. The data that support the findings of this study may be available on reasonable request from the corresponding author [AH].

## Abbreviations

HNC: Head and neck cancer
CPS: Clinical prediction of survival
AS: Actual survival
QoL: Quality of life
QoD: Quality of death
GP: General practitioner
SPSS: Statistical Package for the Social Sciences
BMI: Body Mass Index
ACE-27: Adult Comorbidity Evaluation 27
